# Three-Year Outcomes and Safety of the XEN63 Gel Stent: A Multicenter Prospective Study in Primary Open-Angle Glaucoma

**DOI:** 10.3390/jcm15093528

**Published:** 2026-05-05

**Authors:** José María Martínez-de-la-Casa, María Teresa Marcos-Parra, Elena Millá-Griñó, Teresa Laborda, Rafael Giménez, José Manuel Larrosa, Aritz Urcola, Miguel Ángel Teus, Susana Perucho-Martínez

**Affiliations:** 1 Ophthalmology Unit, Department of Ophthalmology and ORL, Faculty of Medicine, Hospital Clinico San-Carlos, Universidad Complutense de Madrid, Instituto de Investigación Sanitaria del Hospital Clínico San-Carlos (IdISSC), Calle del Prof Martín Lagos, s/n, 28040 Madrid, Spain; 2 Ophthalmology Department, Hospital General Universitario de Alicante, 03010 Alicante, Spain; 3 Ophthalmology Department, Hospital Clinic, 08036 Barcelona, Spain; millagrinoelena@gmail.com; 4 Glaucoma Department, Hospital La Arruzafa, 14012 Córdoba, Spain; tlaborda@hospitalarruzafa.com; 5 Ophthalmology Department, Reina Sofia University Hospital, 14004 Córdoba, Spain; gimenezgomez@hotmail.com; 6 Ophthalmology Department, Miguel Servet University Hospital, 50009 Zaragoza, Spain; 7 Ophthalmology Department, Araba University Hospital, 01004 Araba, Spain; 8 Ophthalmology Department, Alcala University Hospital, 28805 Madrid, Spain; 9 Ophthalmology Department, Hospital Universitario de Fuenlabrada, 28942 Fuenlabrada, Spain; sperucho77@gmail.com

**Keywords:** XEN gel stent, primary open-angle glaucoma, bleb forming devices, IOP, success rates

## Abstract

**Purpose**: To evaluate the 3-year effectiveness and safety of the XEN63 Gel Stent, both as a standalone procedure and combined with phacoemulsification, in patients with primary open-angle glaucoma (POAG). **Methods**: This prospective, multicenter, non-randomized study included 85 eyes (85 patients) with medically-uncontrolled POAG. Subjects underwent either XEN63-standalone implantation (n = 46) or combined XEN63 + Phacoemulsification (n = 39). The primary endpoint was the cumulative probability of complete success (IOP ≥ 6 and ≥18 mmHg and reduction in IOP ≥ 20% from baseline without ocular-hypotensive medications) at 36 months. **Results**: At 36-months, the overall surgical success rate was 67.1% (95% CI: 50.8–86.9%), with significant differences between the standalone (78.3%) and combined groups (53.9%; *p* = 0.0173). Complete success was achieved in 45.9% of the total cohort. Preoperative mean IOP decreased significantly from 21.3 ± 4.7 mmHg to 14.3 ± 4.3 mmHg at the last-follow-up visit (LFUV) (*p* < 0.0001). Correspondingly, the mean number of medications was significantly reduced from 2.3 ± 0.8 to 0.7 ± 1.0 (*p* < 0.0001). Multivariable analysis showed that surgical strategy did not significantly influence IOP reduction. Numerical hypotony (IOP < 6 mmHg) occurred in 23.5% of eyes at Day 1 but resolved in 95% of cases by Month 1. One case of hypotonic maculopathy required device explantation. Serious late-onset events included one endophthalmitis (Month 30) and one retinal detachment (Month 26). Secondary needling was required in 8.2% of eyes. **Conclusions**: The XEN63 Gel Stent provided sustained IOP reduction and a significantly decreased medication burden over 36 months. Outcomes remained consistent regardless of whether the stent was implanted as a standalone procedure or combined with cataract surgery.

## 1. Introduction

Glaucoma comprises a heterogeneous group of chronic optic neuropathies characterized by progressive retinal ganglion cell loss and irreversible visual impairment, remaining a leading cause of blindness worldwide [1,2,3]. Reduction of intraocular pressure (IOP) is currently the only proven modifiable risk factor to delay disease progression [4,5]. Although topical therapy and selective laser trabeculoplasty are commonly used as first-line treatments, many patients ultimately require surgical intervention because of inadequate IOP control, medication intolerance, or poor adherence [3,6,7].

Traditionally, trabeculectomy and non-penetrating filtering procedures have represented the gold standard for medically refractory glaucoma, achieving substantial long-term IOP reduction [8,9]. However, their use is limited by potentially severe complications, including hypotony, choroidal detachment, and bleb-related morbidity [10,11]. These limitations have driven the development of minimally invasive glaucoma surgery (MIGS) and minimally invasive bleb-forming surgery (MIBS) techniques, designed to enhance safety while maintaining effective pressure control [12,13].

The XEN Gel Stent (AbbVie Inc., Chicago, IL, USA) is a 6-mm glutaraldehyde cross-linked porcine gelatin implant that facilitates subconjunctival aqueous humor drainage [14,15]. Its mechanism is based on the Hagen–Poiseuille principle, whereby lumen dimensions determine outflow resistance and pressure reduction. Two models are currently available: XEN45 (45-μm lumen) and XEN63 (63-μm lumen) [14,16]. The XEN63 incorporates a larger internal diameter to reduce flow resistance while maintaining a comparable external profile and minimally invasive delivery through a 27-gauge injector [16,17]. Although the device has obtained CE mark approval in Europe and Canada, it is not currently cleared by the U.S. Food and Drug Administration.

Clinical evidence regarding the XEN63 Gel Stent remains limited, largely consisting of small cohorts with short- to intermediate-term follow-up [16,17,18,19,20,21,22,23]. In a previous study conducted by our group, XEN63 implantation—either standalone or combined with phacoemulsification—resulted in significant reductions in IOP and IOP-lowering medications (*p* < 0.0001 for both), with a favorable safety profile at 12 months [21].

Given the increasing adoption of minimally invasive subconjunctival filtration techniques, the long-term performance of the XEN63 implant remains insufficiently characterized. Therefore, the current study aimed to evaluate the effectiveness and safety of the XEN63 Gel Stent, implanted alone or in combination with cataract surgery, in patients with primary open-angle glaucoma over a 3-year follow-up period.

## 2. Materials and Methods

### 2.1. Study Design

We conducted a prospective, multicenter, open-label study involving a consecutive series of patients with medically recalcitrant primary open-angle glaucoma (POAG). Research activities were performed in accordance with the protocol sanctioned by the Ethics Committee of the Hospital Clínico San Carlos in May 2021 (Reference: HCSC-XEN63R1).

The investigation was conducted in accordance with the principles of the International Council for Harmonization Good Clinical Practice guidelines, the Declaration of Helsinki, and all applicable national regulatory requirements, applying the most protective standards for participants. Written informed consent was obtained from all subjects prior to enrollment. All identifiable patient information was anonymized to ensure confidentiality.

### 2.2. Study Population and Eligibility Criteria

Eligible participants were adults (≥18 years) diagnosed with early-to-moderate POAG with insufficient IOP control under medical therapy, defined as treated IOP between 18 and 33 mmHg while receiving one to four ocular hypotensive medications. Additional inclusion criteria comprised a Shaffer angle grade ≥3 in the superonasal quadrant, adequate healthy and mobile conjunctiva in the intended implantation area, and the capacity to provide informed consent and comply with study procedures [22].

Key exclusion criteria included any glaucoma subtype other than POAG, prior incisional glaucoma surgery, intraocular surgery within 3 months before enrollment, conjunctival scarring or pathology in the target quadrant, history of prior corneal procedures; central corneal thickness values ≤490 μm or ≥620 μm; presence of vitreous in the anterior chamber; intraocular silicone oil tamponade; and any active ocular inflammation or infection occurring within 30 days before the surgical intervention, impaired episcleral venous outflow, or hypersensitivity to study-related medications or implant components (including porcine-derived materials or glutaraldehyde) [22].

### 2.3. Surgical Technique

All procedures were performed under local anesthesia. Utilizing an ab interno technique, the XEN63 Gel Stent was positioned within the superior quadrant [17]. To modulate the wound-healing response, an intraoperative subconjunctival injection of mitomycin-C (MMC) (0.1 mL at 0.01–0.02% concentration) was administered. The specific MMC dosage was tailored to each patient’s clinical risk profile and the primary surgeon’s discretion.

Device insertion was performed through a 1.8-mm clear corneal paracentesis. Postoperative management included topical antibiotic and corticosteroid therapy (tobramycin/dexamethasone combination) administered every 2 h during the first postoperative day and gradually tapered over 6–8 weeks.

### 2.4. Follow-Up Schedule

The protocol included one screening visit and one baseline assessment. Postoperative evaluations were scheduled at day 1; week 1 (±2 days); and months 1 (±7 days), 3 (±14 days), 6 (±14 days), 12 (±30 days), 18 (±30 days), 24 (±30 days), 30 (±30 days), and 36 (±30 days).

### 2.5. Outcome Definitions

Outcome measures were defined according to the recommendations of the World Glaucoma Association for glaucoma surgical trials [24]. The primary endpoint was the proportion of eyes achieving complete surgical success at 36 months, accounting for loss to follow-up.

Surgical success was defined as a reduction in IOP ≥20% from baseline with a final IOP between 6 and 18 mmHg. Complete success required achievement of these criteria without ocular hypotensive medications, whereas overall success allowed the use of medications.

Surgical failure was defined by any of the following: IOP >18 mmHg or <20% reduction from baseline on two consecutive visits, IOP <6 mmHg associated with loss of more than two lines of visual acuity, need for additional glaucoma surgery, absence of light perception, or persistent hypotony.

Needling or surgical bleb revision were indicated upon the detection of IOP exceeding individualized targets over two consecutive follow-up visits. Furthermore, interventions were performed in cases of suspected filtration failure, evidenced by bleb fibrosis or encapsulation, which failed to resolve following slit-lamp digital massage or pharmacological management.

### 2.6. Study Groups

Eyes were categorized into two groups: (1) XEN63-standalone, including eyes undergoing isolated XEN63 implantation, and (2) XEN63 + Phaco, including eyes undergoing combined XEN63 implantation and phacoemulsification.

### 2.7. Study Outcomes

The primary endpoint was the cumulative probability of complete surgical success at month 36 using Kaplan–Meier survival analysis.

Secondary endpoints included the overall surgical success rate; mean change in IOP from baseline to month 36; mean IOP at month 36; reduction in the number of ocular hypotensive medications; identification of preoperative and postoperative predictive factors associated with overall and complete success; and the incidence of intraoperative and postoperative adverse events.

### 2.8. Statistical Analysis

Data processing and all statistical computations were executed with MedCalc^®^ Statistical Software (v23.4.9; MedCalc Software Ltd., Ostend, Belgium. https://www.medcalc.org; 2026, accessed on 5 April 2026). To prevent unit-of-analysis errors, data were restricted to a single eye per participant. In cases where both eyes of a patient met the study’s eligibility criteria, the investigator selected the surgical eye for inclusion.

The Shapiro–Wilk test was initially applied to verify the normality of continuous data. For variables following a Gaussian distribution, repeated-measures ANOVA was employed to track longitudinal trends in IOP and the required count of hypotensive medications. Non-normally distributed data were alternatively analyzed using the Friedman test. Baseline intergroup comparisons were conducted via the Mann–Whitney U test, while categorical data distributions were assessed using Fisher’s exact test or the chi-square test, as dictated by cell frequencies.

To control for confounding variables, a multivariate analysis of covariance (MANCOVA) with repeated measures was used to compare IOP changes throughout the study between the standalone XEN63 and combined surgery cohorts. This model incorporated the following preoperative parameters as covariates: IOP and number of ocular hypotensive therapy; pachymetry; and MMC dose.

Risk factors for XEN63 device failure were identified using conditional Cox proportional hazards regression. Variables demonstrating an association at a *p*-value ≤ 0.10 during univariate screening were entered into a multivariate model, which utilized a backward elimination technique (selection cutoff of 0.05).

Kaplan–Meier survival analysis was used to estimate cumulative success probabilities, with the log-rank test determining the significance of differences between groups. To preserve longitudinal data integrity, patients who did not complete the 36-month follow-up or were withdrawn for reasons unrelated to surgical failure were treated as censored at their last available assessment.

For all analyses, a two-tailed *p* < 0.05 was established as the threshold for statistical significance.

## 3. Results

### 3.1. Study Sample

A cohort of 85 eyes from 85 individual patients was analyzed. Among these, 46 eyes (54.1%) underwent the XEN63 procedure as a standalone intervention, while 39 eyes (45.9%) received a combined procedure (XEN63 + Phacoemulsification).

### 3.2. Preoperative Demographic and Clinical Characteristics

The study population had a mean age of 71.5 ± 10.0 years, with no significant intergroup differences (*p* = 0.0610). The gender distribution included 38 women (44.7%), and the entire study group (100%) identified as Caucasian. A comprehensive analysis of the main demographic and clinical preoperative parameters is provided in Table 1.

Preoperative IOP was significantly lower in the XEN63 + Phaco group (20.1 ± 4.2 mmHg) compared with theXEN63 standalone group (22.3 ± 4.9 mmHg) (Hodges–Lehmann median difference: −2.0 mmHg; 95% CI: −4.0 to 0.0 mmHg; *p* = 0.0080).

Consistently, the preoperative number of ocular hypotensive medications was also significantly lower in the XEN63 + Phaco group than in the standalone group (Hodges–Lehmann median difference: 0.0 medications; 95% CI: −1.0 to 0.0; *p* = 0.0121).

No statistically significant differences were detected between groups for the remaining baseline variables (Table 1).

### 3.3. Surgical Success

**Figure 1:**
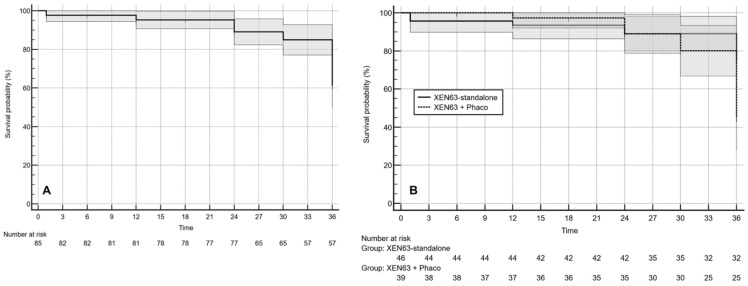
Kaplan–Meier overall survival estimates. The shaded area represents the 95% confidence interval (CI). (**A**) Survival probability analysis for the entire study cohort. (**B**) Kaplan–Meier survival curves depicting surgical success in eyes undergoing XEN63 implantation as a standalone procedure versus combined XEN63 with phacoemulsification (XEN63 + Phaco). The calculated hazard ratio was 2.23 (95% CI: 1.03–4.83; *p* = 0.0420).

The overall surgical success rate was 67.1% (95% CI: 50.8–86.9%) (Figure 1A). When stratified by treatment group, success was achieved in 78.3% (95% CI: 54.8–100.0%) of eyes in the XEN63-standalone group and 53.9% (95% CI: 33.3–82.3%) in the XEN63 + Phaco group, without statistically significant differences between groups (*p* = 0.0173).

**Figure 2:**
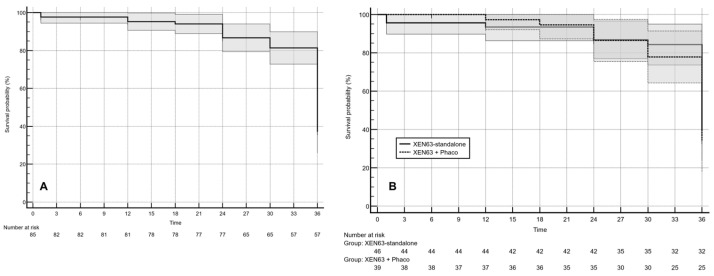
Surgical success and Kaplan–Meier survival analysis based on complete success criteria. The shaded area represents the 95% confidence interval (CI). (**A**) Survival probability analysis in the overall cohort. (**B**) Kaplan–Meier survival curves comparing time to failure between eyes treated with XEN63 as a standalone procedure and those undergoing combined XEN63 implantation with phacoemulsification. No statistically significant difference in survival distribution was observed between groups (hazard ratio: 1.14; 95% CI: 0.64–2.04; *p* = 0.6486). Mean survival time was 33.0 ± 1.2 months (95% CI: 30.6–35.4) in the standalone group and 33.4 ± 0.96 months (95% CI: 31.5–35.3) in the combined-surgery group; the median survival time was 36.0 months in both cohorts.

Complete success was observed in 45.9% (95% CI: 32.6–62.7%) of the overall cohort (Figure 2A), with rates of 47.8% (95% CI: 30.0–72.4%) in the XEN63-standalone group and 43.6% (95% CI: 25.4–69.8%) in the combined-surgery group; these differences were not statistically significant (*p* = 0.7739).

Across the entire study population, surgical failure occurred in 28 eyes (32.9%) (Figure 1A). However, Cox proportional hazards modeling demonstrated a significantly higher risk of failure in the XEN63 + Phaco group compared with the XEN63-standalone group (hazard ratio: 2.23; 95% CI: 1.03–4.83; *p* = 0.0420) (Figure 1B).

Mean survival time was 33.4 ± 1.2 months (95% CI: 31.0–35.9) in the standalone group and 33.8 ± 0.96 months (95% CI: 32.1–35.5) in the combined-surgery group; the median survival time was 36.0 months in both cohorts.

Applying the definition of complete success; namely, an IOP ≥6 and ≤ 18 mmHg with a ≥20% reduction from baseline in the absence of topical ocular hypotensive therapy; the cumulative rate of surgical failure in the overall cohort was 45.9% (Figure 2A). Kaplan–Meier survival modeling revealed no statistically significant difference in failure risk between eyes treated with XEN63 as a standalone procedure and those undergoing combined XEN63 implantation with phacoemulsification (hazard ratio: 1.14; 95% CI: 0.64–2.04; *p* = 0.6486) (Figure 2B).

In the XEN63-standalone group, the estimated mean survival time was 33.0 ± 1.2 months (95% CI: 30.6–35.4 months), with a median survival of 36.0 months. Similarly, in the XEN63 plus phacoemulsification cohort, the mean survival duration was 33.4 ± 0.96 months (95% CI: 31.5–35.3 months), and the median survival was likewise 36.0 months.

### 3.4. Intraocular Pressure

**Figure 3:**
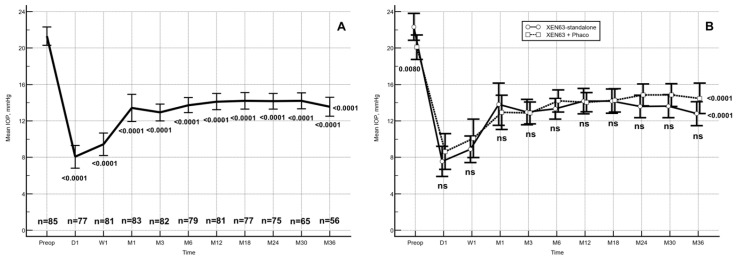
Longitudinal evolution of mean intraocular pressure (IOP) during follow-up. Vertical error bars indicate 95% confidence interval. (**A**) Mean IOP values in the overall study population across all postoperative time points. (**B**) Mean IOP trends in eyes undergoing XEN63 implantation as a standalone procedure and in combination with phacoemulsification (XEN63 + Phaco) throughout follow-up.

Across the full cohort, mean IOP was significantly lowered from 21.3 ± 4.7 mmHg (95% CI: 20.3–22.3) preoperatively to 14.3 ± 4.3 mmHg (95% CI: 13.4–15.2) at the last follow-up visit (LFUV) (Mean IOP lowering: −7.0 mmHg; 95% CI: 18.3 to −5.7 mmHg; *p* < 0.0001. Repeated ANOVA). (Figure 3A). Relative to baseline measurements, IOP values were significantly reduced at every postoperative assessment (*p* < 0.0001 for all comparisons).

In subgroup analyses, both the XEN standalone and XEN combined with phacoemulsification (XEN + Phaco) cohorts exhibited marked IOP decreases. Within the standalone-treatment subgroup, the mean preoperative IOP of 22.3 ± 4.9 mmHg (95% CI: 20.9–23.8) lowered to 13.9 ± 4.2 mmHg (95% CI: 12.4–15.3) at the LFUV visit. Similarly, in the combined-surgery group, mean IOP was reduced from 20.1 ± 3.9 mmHg (95% CI: 18.8–21.5) preoperatively to 14.8 ± 3.7 mmHg (95% CI: 13.6–16.0) at LFUV (*p* < 0.0001 for both groups, repeated-measures ANOVA). Preoperative IOP was significantly greater in the XEN63 standalone cohort, with a Hodges–Lehmann median difference of 2.0 mmHg (95% CI: 0.0–4.0 mmHg; *p* = 0.0080). Nevertheless, no statistically significant intergroup differences in IOP were observed during subsequent follow-up evaluations (Figure 3B). Compared with preoperative values, IOP remained significantly lower at all postoperative time points in both cohorts (*p* < 0.0001 for each time point).

Between-group comparisons at each time point were performed using one-way ANOVA with Scheffé’s post hoc adjustment. Relative to baseline, mean IOP was significantly decreased at every postoperative assessment (*p* < 0.0001), as determined by repeated-measures ANOVA with Greenhouse–Geisser correction.

**Figure 4:**
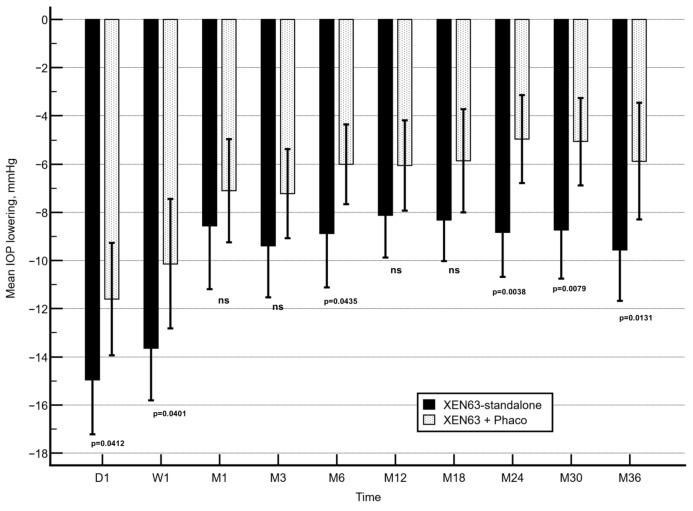
Unadjusted reduction in mean intraocular pressure (IOP) in the XEN63-standalone and XEN63 + Phaco cohorts. Vertical error bars denote the 95% confidence intervals.

The unadjusted mean reduction in IOP was significantly greater in the XEN63-standalone cohort compared with the XEN63 + Phaco group at postoperative day 1, week 1, and at months 6, 24, 30, and 36 (*p* = 0.0412, *p* = 0.0401, *p* = 0.0435, *p* = 0.0038, *p* = 0.0079, and *p* = 0.0131, respectively). At all remaining evaluated follow-up intervals, no statistically significant intergroup differences were identified (Figure 4).

Between-group differences in IOP reduction were assessed using the two-sided Mann–Whitney U test.

However, after multivariable adjustment for potential confounders; including age, baseline IOP, preoperative number of ocular hypotensive agents, central corneal thickness (pachymetry), and MMC dosage; no significant differences in IOP reduction were detected between the two surgical strategies at any assessed time point (Table 2).

### 3.5. Ocular Hypotensive Treatment

A significant reduction in the mean ocular hypotensive medication burden was observed for the entire cohort, declining from 2.3 ± 0.8 preoperatively to 0.7 ± 1.0 at the last follow-up visit (*p* < 0.0001). Subgroup analysis confirmed this trend in both the XEN63-standalone (2.5 ± 0.7 to 0.9 ± 1.0; *p* < 0.000) and XEN63-phacoemulsification groups (2.1 ± 0.9 to 0.5 ± 0.8; *p* < 0.0001). Notably, the magnitude of medication reduction was comparable between groups (−1.7 ± 1.2 vs. −1.6 ± 1.1 agents, respectively), with no statistically significant difference detected (Hodges–Lehmann median difference: 0.0; 95% CI: −1.0 to 1.0; *p* = 0.9964).

### 3.6. Best Corrected Visual Acuity

In the overall study cohort, best corrected visual acuity (BCVA) demonstrated a statistically significant improvement from a preoperative mean of 0.62 (95% CI: 0.55–0.70) to 0.77 (95% CI: 0.70–0.84) at the LFUV (*p* = 0.0014).

Subgroup analysis revealed no significant change in BCVA between baseline and LFUV in the XEN63-standalone group (mean difference: 0.00; 95% CI: −0.14 to 0.12; *p* = 0.8814). In contrast, eyes undergoing combined XEN63 implantation with phacoemulsification exhibited a significant gain in BCVA from baseline to month 12 (mean difference: 0.26; 95% CI: 0.15–0.38; *p* < 0.0001).

### 3.7. Risk Factors

Cox proportional-hazards regression analysis revealed that the treatment procedure was the only factor significantly associated with surgical success under the overall success criteria. Specifically, combined surgery was associated with a significantly lower hazard of success compared to standalone procedures (Hazard Ratio [HR]: 0.39; 95% CI: 0.17–0.87; *p* = 0.042). Conversely, no factors reached statistical significance when evaluated against the complete success criteria. Detailed results for all analyzed risk factors across both success criteria are presented in Table 3.

### 3.8. Safety Outcomes

With respect to safety, early postoperative numerical ocular hypotony (IOP < 6 mmHg) was observed in 20 eyes (23.5%) on the first postoperative day. In 19 of these cases, IOP normalized spontaneously without long-term consequences by postoperative month 1. At month 3, persistent hypotony was documented in 2 eyes (2.4%), whereas no cases were detected at month 6. The hypotony episodes were subclinical and not associated with maculopathy in all but one case. In that eye, hypotonic maculopathy necessitated implant explantation at month 1. Two months following device removal, BCVA improved to baseline levels (0.4).

Apart from hypotony, the most frequently reported postoperative adverse events included shallow anterior chamber (8/85 eyes), corneal dellen (4/85 eyes), hyphema (4/85 eyes), choroidal detachment (2/85 eyes), vitreous wick (1/85 eyes), and fibrin formation in the anterior chamber (1/85 eyes). All these events were mild in severity and resolved with conservative medical management. One eye developed endophthalmitis at month 30, ultimately requiring evisceration. Additionally, one patient underwent pars plana vitrectomy for retinal detachment 26 months after surgery.

Regarding secondary interventions, 7 eyes (8.2%) required needling procedures, and 5 eyes (5.9%) underwent surgical bleb revision. Device-related complications included one case (1.2%) of implant replacement due to intraoperative damage during needling and one case of device explantation secondary to hypotonic maculopathy (Table 4).

## 4. Discussion

This prospective, multicenter study evaluated the long-term efficacy and safety of the XEN63 Gel Stent in patients with POAG over a 36-month follow-up period. Our results demonstrated that XEN63 implantation, whether performed as a standalone procedure or combined with phacoemulsification, achieved sustained and clinically meaningful IOP reductions, accompanied by a significant decrease in the need for ocular hypotensive medication throughout the three years follow-up period.

The cumulative probability of overall surgical success at 36 months was 67.1% (95% CI: 50.8–86.9%), while complete success was achieved in 45.9% of eyes (95% CI: 32.6–62.7%). Although crude comparisons demonstrated a statistically significant difference in overall success between standalone and combined procedures (*p* = 0.0173), no significant difference was observed for complete success (*p* = 0.7739). Moreover, cox proportional hazards modeling indicated that combined surgery was associated with a lower probability of achieving overall success (HR: 0.39; 95% CI: 0.17–0.87; *p* = 0.042). This association was not observed when the stricter definition of complete success was applied. These findings indicate that, despite comparable point estimates of success, time-to-failure dynamics differed between surgical strategies, with a significantly higher risk of failure observed in the combined procedure group. This finding may be attributed to the heightened intraocular inflammation and altered cytokine profile induced by concomitant phacoemulsification, which can accelerate the subconjunctival fibrotic response and potentially compromise bleb longevity [25].

Direct comparison with the literature is limited by the scarcity of long-term data on the current XEN63 model. The most relevant benchmark remains our previously published 12-month results [21], in which the overall success rate was 68.8% (55/80 eyes), with no significant differences between standalone and combined procedures (*p* = 0.6133). Importantly, these figures are consistent with the 36-month outcomes reported herein, indicating stability of the surgical effect beyond the first postoperative year. The absence of a substantial decline in success rates over time supports the durability of the pressure-lowering effect achieved with XEN63 implantation.

Longer follow-up data extending to 4 and 5 years are available only for earlier iterations of the XEN63 device [26,27], which may limit direct comparability with the current platform. In those studies, complete success, defined as postoperative IOP ≥ 6 and ≤18 mmHg with at least a 20% reduction from baseline and no medication, was achieved in 22.6% (12/53) and 27.3% (3/11) of eyes, respectively. These rates are numerically lower than the 45.9% complete success observed in our cohort at 36 months, potentially reflecting refinements in device design, surgical technique, patient selection, or perioperative management.

Fea et al. [18] in a retrospective real-world series of 23 eyes followed for 18 months, reported a significant reduction in mean IOP from 27.0 ± 7.8 mmHg to 14.1 ± 3.4 mmHg (*p* < 0.0001), together with a decrease in medication burden. Similarly, Voykov et al. [20] in a prospective case series of six eyes, described a marked decline in median IOP from 35.5 mmHg to 11.5 mmHg at 24 months, with most patients achieving complete success. Although baseline IOP levels were higher in those studies, the magnitude and persistence of IOP reduction are broadly consistent with our findings, reinforcing the effectiveness of the device across different clinical settings.

In our cohort, mean IOP decreased by 7.0 ± 6.0 mmHg (95% CI: 5.7 to 8.3; *p* < 0.0001). Both surgical strategies were associated with significant pressure reduction. The standalone group showed a greater unadjusted mean IOP decrease than the combined group; however, after adjustment for potential confounders; including age, baseline IOP, preoperative medication burden, central corneal thickness, and MMC dosage; these differences were no longer statistically significant. Overall, both approaches provided comparable long-term IOP control.

The reduction in the number of glaucoma medications observed at 36 months is consistent with previous short- and intermediate-term studies of XEN63 [16,17,18,19,20,21,22,23], suggesting sustained pharmacologic independence in a substantial proportion of patients.

From a safety perspective, the device exhibited an acceptable profile. Early postoperative hypotony (IOP < 6 mmHg) occurred in 23.5% of eyes on postoperative day 1, resolving spontaneously by month 1 in 95% of cases. With one exception, these episodes were numerical hypotony without associated maculopathy. The single case of clinically significant hypotony required implant removal; visual acuity subsequently returned to baseline. The incidence observed in our study is comparable to previously reported rates [17,18,19] and appears lower than those described by Voykov et al. [20] and Bertolani et al. [22]. When contextualized against data for the XEN45 device, where hypotony has been reported in approximately 20% of cases [28], our findings remain within the expected range for subconjunctival filtration procedures. The 23.5% incidence of early numerical hypotony in this study exceeds the 9.59% typically reported for the XEN45 [29]. This higher rate is likely attributable to the lower filtration resistance inherent to the XEN63’s larger internal diameter. However, similar to the XEN45, the majority of these cases were transient and re-solved without surgical re-intervention, mirroring the established safety profile regarding the rarity of chronic hypotony.

Serious adverse events were rare and occurred late in follow-up. One case of endophthalmitis at month 30 required evisceration, and one patient underwent pars plana vitrectomy for retinal detachment at 26 months. These events were not attributed to the mechanical properties of the shunt itself but rather to the perioperative environment or underlying ocular pathology. Secondary interventions were relatively infrequent: needling was performed in 8.2% of eyes, a lower rate than that reported by Sacchi et al. [23] and 5.9% required surgical bleb revision. Device replacement was necessary in two cases. The 1.2% incidence of endophthalmitis resulting in evisceration, while clinically significant, is consistent with rates reported in landmark glaucoma surgery trials. The Tube Versus Trabeculectomy Study documented a cumulative endophthalmitis incidence of approximately 1% over five years [11]. Similarly, a recent systematic review of XEN gel stent complications reported rates ranging from 0.4% to 3.0%, situating our finding (1.2%) within the expected safety profile for subconjunctival filtering procedures [28]. Although the clinical outcome in this case was severe, the observed incidence does not constitute a statistical outlier within the context of bleb-forming glaucoma surgeries.

Several limitations should be acknowledged. The study was non-randomized, open-label, and lacked a control group, although outcome assessment was conducted in a masked manner. Baseline IOP differed between treatment groups; however, statistical adjustment using ANCOVA mitigated the potential impact of this imbalance. This study was also limited by its single-arm design and the absence of a direct comparator, which limits the ability to account for selection bias. Additionally, the cohort consisted exclusively of Caucasian patients with POAG, which may limit generalizability to other populations or glaucoma subtypes. Finally. A 30% attrition rate at 3 years constitutes a relevant limitation and introduces the potential for attrition bias. Such dropout levels are common in long-term prospective glaucoma surgery studies, where patients may require adjunctive (“rescue”) therapy or additional surgical interventions, leading to exclusion from primary success analyses. Nevertheless, the resulting loss of data reduces the statistical power of long-term secondary endpoints. The reduced sample size at 36 months warrants cautious interpretation of per-protocol outcomes. However, the baseline comparability between retained participants and those lost to follow-up supports the representativeness of the analyzed cohort. Future studies with larger initial sample sizes and structured retention strategies are needed to confirm the long-term outcomes associated with the XEN63 device. Despite these limitations, the 36-month prospective, multicenter design represents a notable strength and provides valuable long-term data on the performance of the current XEN63 device.

## 5. Conclusions

In this prospective, multicenter study, XEN63 implantation provided sustained intraocular pressure reduction and significant medication sparing over 36 months in patients with primary open-angle glaucoma. While both cohorts demonstrated significant postoperative improvements, the combined procedure was associated with a significantly higher hazard of failure compared to standalone surgery. After adjustment for confounding factors, long-term outcomes remained similar between groups. The safety profile was acceptable, with most adverse events being transient and manageable. Overall, these findings support XEN63 as an effective and durable surgical option for long-term intraocular pressure control in appropriately selected patients.

## Figures and Tables

**Table 1:** Main preoperative demographic and clinical characteristics of the study population.

	Overall (*n* = 85)	XEN Alone (*n* = 46)	XEN + Phaco (*n* = 39)	*p* ^a^
Age, years				0.0610
Mean (SD)	71.5 (10.0)	73.4 (9.5)	69.3 (10.3)	
Median (IqR)	72.0 (66.0 to 78.0)	74.0 (69.0 to 78.8)	71.0 (66.0 to 75.8)	
Sex, n (%)				0.8299 ^b^
Women	38 (44.7)	20 (43.5)	18 (46.2)	
Men	47 (55.3)	26 (56.5)	21 (53.8)	
Race, n (%)				1.0000 ^b^
Caucasian	85 (100.0)	46 (100.0)	39 (100.0)	
Eye, n (%)				0.0836 ^b^
Right	44 (51.8)	28 (60.9)	16 (41.0)	
Left	41 (48.2)	18 (39.1)	23 (59.0)	
Comorbidities *, n (%)				0.9695 ^c^
None	44 (51.8)	21 (45.7)	23 (59.0)	
HBP	17 (20.0)	9 (19.6)	8 (20.5)	
Dyslipidemia	25 (29.4)	12 (26.1)	13 (33.3)	
DM	11 (12.9)	6 (13.0)	5 (12.8)	
CVD	6 (7.1)	4 (8.7)	2 (5.1)	
Respiratory diseases	3 (3.5)	1 (2.2)	2 (5.1)	
Other	15 (17.7)	7 (15.2)	8 (20.5)	
Glaucoma type, n (%)				1.0000 ^b^
POAG	85 (100.0)	46 (100.0)	39 (100.0)	
Preoperative IOP, mmHg				0.0080
Mean (SD)	21.3 (4.7)	22.3 (4.9)	20.1 (4.2)	
Median (IqR)	20.0 (18.0 to 24.0)	22.0 (19.0 to 24.0)	19.0 (18.0 to 21.8)	
NOHM, n				0.0121
Mean (SD)	2.3 (0.8)	2.5 (0.7)	2.1 (0.9)	
Median (IqR)	2.0 (2.0 to 3.0)	3.0 (2.0 to 3.0)	2.0 (1.0 to 3.0)	
NOHM, n (%)				0.0382 ^c^
1	14 (16.5)	3 (6.5)	11 (28.2)	
2	35 (41.2)	19 (41.3)	16 (41.0)	
3	31 (36.5)	21 (45.7)	10 (25.6)	
4	5 (5.9)	3 (6.5)	2 (5.1)	
Pachymetry, µm				0.3407
Mean (SD)	533.2 (35.5)	536.8 (34.2)	529.0 (38.8)	
Median (IqR)	532.5 (514.3 to 551.0)	532.8 (520.0 to 555.0)	532. 0 (510.3 to 548.0)	
BCVA **				0.0993
Mean (SD)	0.62 (0.27)	0.69 (0.27)	0.55 (0.27)	
Median (IqR)	0.60 (0.40 to 0.80)	0.70 (0.50 to 0.80)	0.60 (0.40 to 0.80)	
VF damage, dB				0.0959
MD				
Mean (SD)	−6.33 (3.73)	−7.30 (4.08)	−5.46 (3.18)	
Median (IqR)	−5.94 (−8.67 to −3.30)	−6.87 (−11.55 to −3.97)	−4.84 (−8.08 to −3.26)	

^a^ Two-tailed Mann-Whitney U test. ^b^ Fisher exact test. ^c^ Chi-squared test. * One patient may have more than one comorbidity. ** Snellen. SD: Standard deviation; IqR: Interquartile range; POAG: primary open-angle glaucoma; NOHM: Number of ocular hypotensive medication; BCVA: Best corrected visual acuity; VF: Visual field; MD: Mean defect; HBP: High blood pressure; DM: Diabetes mellitus; CVD: Cardiovascular disease; Phaco: Phacoemulsification.

**Table 2:** Adjusted mean change in intraocular pressure (IOP) relative to baseline in the XEN63-standalone and XEN63 combined with phacoemulsification cohorts. The multivariable regression analysis included the surgical strategy (XEN63 standalone vs. XEN63 combined with phacoemulsification) as the principal independent variable. Adjustments were made for potential confounders, including patient age, baseline intraocular pressure, number of preoperative ocular hypotensive agents, central corneal thickness (pachymetric measurement), and the intraoperatively administered dose of mitomycin C.

	Mean IOP Lowering			
	XEN63-Standalone	XEN63 + Phaco	Difference	*p* ^a^
Day1				0.2387
n	41	36		
Mean (SE)	−14.2 (0.9)	−12.5 (1.0)	−1.7 (1.4)	
95% CI	−16.0 to −12.4	−14.5 to −10.6	−4.4 to 1.1	
Week 1				0.3314
n	43	38		
Mean (SE)	−12.6 (0.9)	−11.3 (1.0)	−1.3 (1.4)	
95% CI	−14.4 to −10.9	−13.2 to −9.4	−4.1 to 1.4	
Month 1				0.5857
n	45	38		
Mean (SE)	−7.5 (1.1)	−8.4 (1.2)	0.9 (1.7)	
95% CI	−9.6 to −5.4	−10.7 to −6.1	−2.4 to 4.2	
Month 3				0.5773
n	43	39		
Mean (SE)	−8.6 (0.7)	−8.1 (0.7)	−0.5 (1.0)	
95% CI	−9.9 to −7.3	−9.4 to −6.7	−2.5 to 1.4	
Month 6				0.3285
n	42	37		
Mean (SE)	−8.0 (0.6)	−7.1 (0.6)	−0.9 (0.9)	
95% CI	−9.2 to −6.8	−8.3 to −5.8	−2.7 to 0.9	
Month 12				0.7524
n	44	37		
Mean (SE)	−7.3 (0.6)	−7.0 (0.7)	−0.3 (1.0)	
95% CI	−8.6 to −6.1	−8.4 to −5.6	−2.2 to 1.6	
Month 18				0.6539
n	42	35		
Mean (SE)	−7.4 (0.7)	−7.0	−0.4 (1.0)	
95% CI	−8.7 to −6.1	−8.4 to −5.5	−2.5 to 1.6	
Month 24				0.0854
n	41	34		
Mean (SE)	−7.8 (0.6)	−6.2 (0.7)	−1.6 (0.9)	
95% CI	−9.0 to −6.6	−7.5 to −4.9	−3.4 to 0.2	
Month 30				0.0581
n	34	31		
Mean (SE)	−7.9 (0.6)	−6.0 (0.7)	−1.9 (1.0)	
95% CI	−9.1 to −6.6	−7.4 to −4.7	−3.8 to 0.1	
Month 36				0.0569
n	31	25		
Mean (SE)	−8.9 (0.7)	−6.7 (0.8)	−2.2 (1.1)	
95% CI	−10.3 to −7.5	−8.3 to −5.1	−4.4 to 0.1	

^a^ analysis of covariance ANCOVA. n: Number of eyes; IOP: Intraocular pressure; SE: Standard error; CI: Confidence interval; Phaco: Phacoemulsification.

**Table 3:** Factors influencing the probability of surgical failure: A Cox proportional-hazards model. Data represent the univariate and multivariate regression analyses for 80 eyes, evaluating the predictive value of both preoperative and post-intervention parameters.

Surgery Failure (Overall Success)		
Variable	Univariable	
	HR (95% CI)	*p*
Age, per additional year	1.00 (0.96–1.04)	0.9225
Sex, female	1.35 (0.64–2.85)	0.4250
Surgery, XEN + Phaco	2.23 (1.03–4.83)	0.0420
MMC concentration, 0.02%	0.92 (0.42–2.04)	0.8459
Preoperative IOP, per mmHg increased	0.94 (0.86–1.03)	0.1837
Preoperative NOHM, per additional medication	1.05 (0.65–1.68)	0.8531
Day 1 IOP, per mmHg increased	0.99 (0.93–1.07)	0.8440
Week 1 IOP, per mmHg increased	1.02 (0.96–1.09)	0.5809
Day 1 IOP lowering, per mmHg lowered	1.01 (0.97–1.06)	0.5582
Week 1 IOP lowering, per mmHg lowered	1.02 (0.98–1.08)	0.2890
Surgery failure (Complete success)		
Variable	Univariable	
	HR (95% CI)	*p*
Age, per additional year	1.00 (0.96–1.03)	0.7811
Sex, female	1.16 (0.65–2.06)	0.6198
Surgery, XEN + Phaco	1.14 (0.64–2.04)	0.6486
MMC concentration, 0.02%	0.84 (0.45–1.58)	0.5925
Preoperative IOP, per mm Hg increased	0.98 (0.92–1.05)	0.5562
Preoperative NOHM, per additional medication	1.07 (0.74–1.55)	0.7129
Day 1 IOP, per mmHg increased	0.99 (0.94–1.05)	0.7248
Week 1 IOP, per mmHg increased	1.01 (0.96–1.06)	0.6700
Day 1 IOP lowering, per mmHg lowered	1.00 (0.97–1.04)	0.9376
Week 1 IOP lowering, per mmHg lowered	1.01 (0.97–1.05)	0.6173

HR = Hazard ratio; CI = Confidence interval; Phaco: Phacoemulsification; MMC: Mitomycin-C; IOP = Intraocular pressure; NOHM: Number of ocular hypotensive medications.

**Table 4:** Postoperative complications.

Complication, n	Day1	Week1	Month1	Month3	Month6	Month12	Month24	Month36
Transient Hypotony *	20	18	0	2	0	0	0	0
Hypotonic maculopathy	0	0	1	0	0	0	0	0
IOP Spikes **	3	1	0	0	0	0	0	0
Hyphema	3	1	0	0	0	0	0	0
Corneal Dellen	0	3	1	0	0	0	0	0
AC Blood ***	1	1	0	0	0	0	0	0
Maculopathy	0	1	0	0	0	0	0	0
Shallow AC	5	2	1	0	0	0	0	0
Bleb leakage	1	0	0	0	0	0	0	0
Choroidal detachment	1	1	0	0	0	0	0	0
Retinal detachment ^1^	0	0	0	0	0	0	0	1
Vitreous wick	0	1	0	0	0	0	0	0
Fibrin in AC	0	1	1	0	0	0	0	0
Endophthalmitis ^2^	0	0	0	0	0	0	0	1
Surgical revision	0	0	4	0	0	0	0	0
XEN removal	0	0	1 ^†^	0	1	0	0	0

* Subclinical. ** Intraocular pressure > 30 mmHg. *** Approximately ½ of the anterior chamber. ^†^ Due to hypotonic maculopathy. ^1^ At month 26. Retinal detachment was successfully resolved with surgery. ^2^ At month 30. The eye was eviscerated. AC: Anterior chamber.

## Data Availability

The datasets used and/or analyzed during the current study available from the corresponding author on reasonable request.

## Abbreviations

The following abbreviations are used in this manuscript:

BCVA	best corrected visual acuity
IOP	Intraocular pressure
LFUV	Last-follow-up visit
MIBS	Minimally invasive bleb-forming surgery
MIGS	Minimally invasive glaucoma surgery
MMC	Mitomycin-C
POAG	Primary open-angle glaucoma
